# Selective cytotoxic effects of nitrogen-doped graphene coated mixed iron oxide nanoparticles on HepG2 as a new potential therapeutic approach

**DOI:** 10.1186/s11671-024-03977-y

**Published:** 2024-02-22

**Authors:** Zeynep Demir, Berkay Sungur, Edip Bayram, Aysun Özkan

**Affiliations:** 1 Department of Biology, Institute of Natural and Applied Sciences, Akdeniz University, 07070 Antalya, Turkey; 2 Department of Chemistry, Institute of Natural and Applied Sciences, Akdeniz University, 07070 Antalya, Turkey; 3 Department of Chemistry, Faculty of Science, Akdeniz University, 07070 Antalya, Turkey; 4 Department of Biology, Faculty of Science, Akdeniz University, 07070 Antalya, Turkey

**Keywords:** Hepatoma G2 cells, Nitrogen-doped graphene, Iron oxide nanoparticles, Cytotoxicity, Selective therapeutics

## Abstract

New selective therapeutics are needed for the treatment of hepatocellular carcinoma (HCC), the 7th most common cancer. In this study, we compared the cytotoxic effect induced by the release of pH-dependent iron nanoparticles from nitrogen-doped graphene-coated mixed iron oxide nanoparticles (Fe_x_O_y_/N-GN) with the cytotoxic effect of nitrogen-doped graphene (N-GN) and commercial graphene nanoflakes (GN) in Hepatoma G2 (HepG2) cells and healthy cells. The cytotoxic effect of nanocomposites (2.5–100 ug/ml) on HepG2 and healthy fibroblast (BJ) cells (12–48 h) was measured by Cell Viability assay, and the half maximal inhibitory concentration (IC_50_) was calculated. After the shortest (12 h) and longest incubation (48 h) incubation periods in HepG2 cells, IC_50_ values of Fe_x_O_y_/N-GN were calculated as 21.95 to 2.11 µg.mL^−1^, IC_50_ values of N-GN were calculated as 39.64 to 26.47 µg.mL^−1^ and IC_50_ values of GN were calculated as 49.94 to 29.94, respectively. After 48 h, Fe_x_O_y_/N-GN showed a selectivity index (SI) of 10.80 for HepG2/BJ cells, exceeding the SI of N-GN (1.27) by about 8.5-fold. The high cytotoxicity of FexOy/N-GN was caused by the fact that liver cancer cells have many transferrin receptors and time-dependent pH changes in their microenvironment increase iron release. This indicates the potential of Fe_x_O_y_/N-GN as a new selective therapeutic.

**Graphical abstract:**

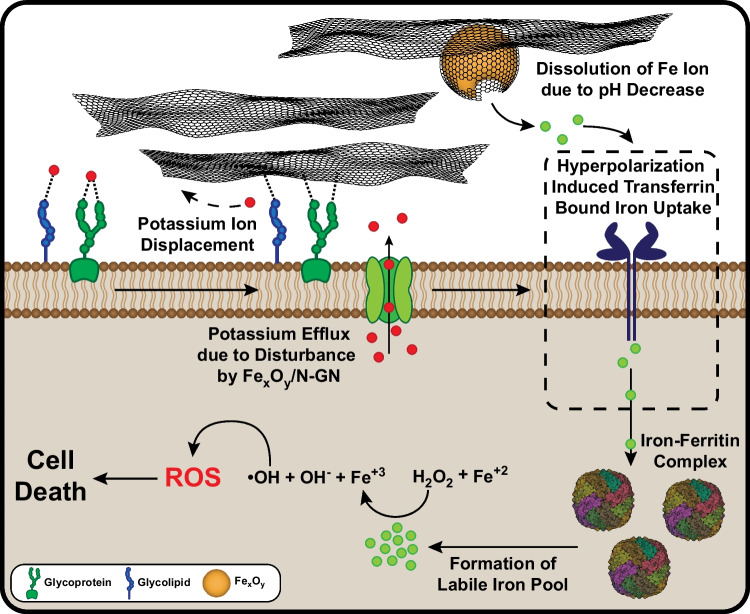

**Supplementary Information:**

The online version contains supplementary material available at 10.1186/s11671-024-03977-y.

## Introduction

Hepatocellular carcinoma (HCC) is the 7th most common cancer type which has a poor prognosis as many others. Although a variety of treatment approaches have been developed, most of the traditional methods, such as surgery, chemotherapy, and radiation therapy, have limited effectiveness in inhibiting tumor growth and often suffer from significant side effects. As a result, there is a dire need for new treatment strategies. Recently, scientists have been exploring the use of functionalized nanomaterials in nanomedicine for cancer treatment. As one of the promising materials, iron oxide nanoparticles have been extensively investigated for their potential applications in chemotherapy, and drug delivery systems through their magnetic properties or phototherap[1]. However, the potential toxicity of these iron oxide nanoparticles should not be overlooked despite their innovative beneficial applications and characteristics.

Studies have shown that high doses of these iron oxide nanoparticles can lead to animal death through rapid hemolysis, while lower doses can cause oxidative stress and cell damage by generating hydroxyl radicals. Ultrasmall iron oxide nanoparticles, in particular, have been found to be highly toxic and lethal to animals at a dosage of 100 mg/kg. Although significant toxicity has been observed in cancer cells, the full extent of iron oxide nanoparticle toxicity in humans remains unclear [2–7]. Therefore, many studies focused on creating composites of these iron oxide nanoparticles, with carbon-based materials. Among carbon-based materials, graphene and its derivatives were commonly chosen due to their modular physicochemical, mechanical, and electronic capabilities with heteroatom doping and their existing applications in biomedical studies [8–11]. However, despite the inertness of graphene-based materials, their cytotoxic effects are also worthy of extensive investigation.

**Fig. 1:**
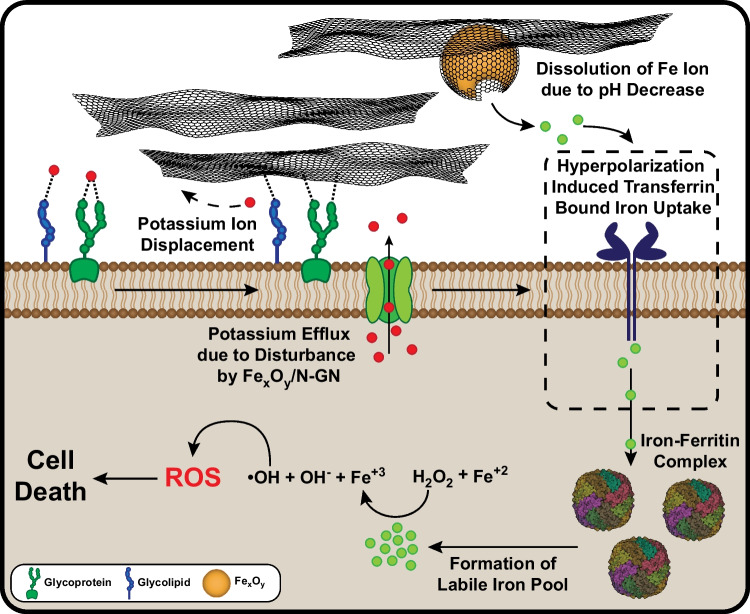
Schematic representation of the cytotoxic effect of Fe_x_O_y_/N-GN

Therefore, in this study, a nitrogen-doped graphene-coated mixed iron oxide composite material (Fe_x_O_y_/N-GN) was investigated as a potential new therapeutic agent, and nitrogen-doped graphene nanoflakes (N-GN) and commercial graphene nanoflakes (GN) were chosen as both reference materials and therapeutic agent. As expected, the release of the iron ion from iron oxide nanoparticles is pH dependent and cancer cells typically have an acidic pH between 5.6 and 6.8 due to their metabolic characteristics, hypoxia, and poor blood perfusion. This acidic microenvironment can lead to the development of a smart cancer nanotherapeutic approach. This study aims to investigate the cytotoxicity effect of Fe_x_O_y_/N-GN by pH-dependent and controlled release of ionic iron atoms on HepG2, a commonly used cancer cell line in studies of iron metabolism, transferrin receptors, and cell death induced by metal oxide nanoparticles as depicted in Fig. 1. When considering the dose and time factors along with nitrogen-doped graphene coating for controllable dissolution, Fe_x_O_y_/N-GN would only show a cytotoxic nature in the cancer microenvironment rather than in healthy tissue. We believe that the results of this study will contribute to the identification of new cancer treatment strategies and provide insights into the differential responses of cancer cells and healthy cells to Fe_x_O_y_/N-GN, adding to the existing literature [12].

## Materials and methods

### Materials

The chemicals used in experiments were all high-purity grade and used without further need for purification. DMF (99.8%, CAS: 68–12–2), Na (99.9%, CAS: 7440–23–5), HCl (37%, CAS: 7647–01–0), K_4_[Fe(CN)_6_] (≥ 98.5%, CAS: 14,459–95–1), and K_3_[Fe(CN)_6_] (≥ 99.0%, CAS: 13,746–66-2), were supplied from Sigma-Aldrich. The commercial graphene was purchased from Nanografi (Türkiye) and used as a reference sample. Ultrapure water (18.2 MΩ) from the Milli-Q UV water purification system was used as needed.

### Solvothermal synthesis procedure

The procedure for the synthesis of N-GN and Fe_x_O_y_/N-GN by a solvothermal method has been described in detail previously [13, 14]. To produce Fe_x_O_y_/N-GN, K_4_[Fe^II^(CN)_6_] (1.5 g) and K_3_[Fe^III^(CN)_6_] (1.5 g) precursors were added as iron sources to the initial Na and DMF precursors and followed by the same procedure for N-GN.

### Characterization of samples

Morphological properties were analyzed by using FEI QUANTA FEG 250 (10 kV) scanning electron microscope (SEM) with energy-dispersive X-ray spectroscopy (EDS). Powder XRD patterns were recorded at 2θ = 10–90^◦^ by using an X-ray diffractometer (BRUKER D8 ADVANCED TWIN-TWIN) using Cu Kα radiation (λ = 1.54056 Å) operating at 40 kV/30 mA. Raman spectra of powder samples were acquired using Renishaw inVia Raman microscope with 514 nm excitation of an argon ion laser. Surface elemental composition and bonding configurations of samples were determined with X-ray photoelectron spectroscopy (XPS) measurements by using Thermo Fisher K-Alpha (see SI for details). Shirley's method was used for baseline correction, while Gaussian distribution functions were applied to deconvolution of XPS. Zeta potentials and hydrodynamic diameters of the samples were measured by dynamic light scattering (DLS) technique with Malvern Zeta sizer Nano series Nano-ZS device [13, 14].

### Cell lines

The Hepatoma G2 (HepG2) and human healthy fibroblast (BJ) cell lines (purchased from the American Type Culture Collection) were used in this study. HepG2 cells were grown in Minimum Essential Medium (MEM) (Lot No: 1109206), and BJ cells were grown in Dulbecco's Modified Eagle Medium (DMEM) (Lot No: 2418665) medium supplemented with 10% (v/v) fetal bovine serum (Lot No: C4343) and 1% (v/v) antibiotic–antimycotic solution (Lot No: 1864851) in a humidified atmosphere containing 5% CO_2_ at 37 °C. For subculturing, cells were harvested after trypsin/ethylenediaminetetraacetic acid (Lot No: 1302763) treatment at 37 °C. Cells were used when monolayer confluence had reached 75% [15].

### Cytotoxicity assay

The cytotoxicity assay procedure followed the OECD (Document: 46,754,707) guideline. The cells were seeded into 96-well microplates (1 × 10^4^ cells well) for 24 h and then treated with different concentrations of the GN, N-GN, Fe_x_O_y_/N-GN (2.5–100 μg/mL) for 12, 24, 36, and 48 h. The appropriateness of the range of concentrations was reached after preliminary experiments. Cytotoxicity of the graphene derivatives was assayed by Cell Titer-Blue® Cell Viability Assay Kit (Lot No: 0000107812). The assay is based on the ability of live cells to convert a redox dye (resazurin) into a fluorescent product (resorufin). Nonviable cells rapidly lose metabolic capacity and thus do not generate a fluorescent signal. Following cellular reduction, fluorescence is recorded at 560 nm excitation / 590 nm emissions using PerkinElmer LS55 [16]. The data were expressed as average values obtained from eight wells for each concentration. IC_50_ values were calculated using two parameters Hill (Eq. 1) and Linear models, respectively. Graphene derivatives were dissolved in water.

1 $$\text{Cell Viability (\%) = }\frac{100}{\text{1} + {\left(\frac{\text{Dose}}{{\text{IC}}_{50}}\right)}^{\text{Hill Coefficient}}}$$

The selectivity index (SI) is defined as the ratio of the toxic sample concentration to the bioactive concentration [17, 18]. The selectivity index (SI) was calculated according to Eq. 2 to determine the therapeutic efficiency of the graphene samples.

2 $$\text{Selectivity Index }\left({\text{SI}}\right)\text{ = }\frac{{\text{IC}}_{50}^{\text{BJ}}}{{\text{IC}}_{50}^{\text{HepG2}}}$$

### Determination of 8-hydroxy-2′-deoxyguanosine level

Cells were plated at a density of 10 × 10^5^ cells/well (100 mm) and incubated with GN, N-GN, and Fe_x_O_y_/N-GN at IC_50_ concentration for 24 and 48 h. After DNA purification from cultured cells (Genomic DNA Mini Kit, Invitrogen), genomic DNA samples were used to quantify 8-hydroxy-2′-deoxyguanosine (8-OHdG) with a competitive ELISA kit (8-OHdG Check New (High Sensitivity), Japan Institute for Control of Aging, Fukuroi, Japan). Microtiter ELISA plates were pre-coated with 8-OHdG. Fifty microlitres of sample and primary antibody were added to each well and incubated overnight at 4 °C. The wells were washed three times and then 100 μL of secondary antibody was added to each well and the wells were incubated for 1 h at room temperature. The wells were washed three times again. After that, enzyme–substrate solutions were added, and the wells were incubated at room temperature for 15 min. The reaction was stopped by adding the termination solution. The absorbance was read at a wavelength of 450 nm [19].

### ***Fe ion dissolution from Fe***_***x***_***O***_***y***_***/N-GN***

To investigate the pH-dependent dissolution kinetics, Fe_x_O_y_/N-GN was dissolved in saline solutions (NaCl 0.137 M, KCl 0.0027 M) at pH 7 and 6. Each solution was incubated for 48 h at 200 rpm and 24 ℃. At specific periods, 3 mL of suspension was taken and filtered through a 0.45 µm syringe filter. Iron content was analyzed with the modified version of the procedure in the study of Turrina et.al [20]. 160 µL filtered suspensions were mixed with 4µL NH3OH.HCl (100 g/L), 20 µL 1,10-Phenanthroline (1 g/L), and 16 µL Acetate Buffer (0.1 M, pH 4.5) to form a red–orange complex between Fe^2+^ and 1,10-Phenanthroline. The absorbance of the 200 µL solutions was taken at 508 nm with MicroTiter ELISA. A calibration curve was prepared with 0, 5, 10, 15, 20, 25, 30, 35, 50, 45, 50, 75, 100, 125, and 150 μL of 0.0002 M Fe^2+^ stock solutions with the same procedure above. All the solutions used in the calibration curve were filled up to 200 µL with deionized water.

### Data analysis

All cytotoxic values of two independent experimental groups with three replicates were given as the mean. Statistical analysis of the data was evaluated using the SPSS (v.23) program and MATLAB (R2022a) Curve Fitting App. ANOVA-GLM (General Linear Model) and pairwise The Tukey Multiple Comparison Test were used to determine the relationships between cytotoxic experimental groups and significance levels in statistical analysis, respectively.

## Results and discussion

### Physicochemical characterization

**Fig. 2:**
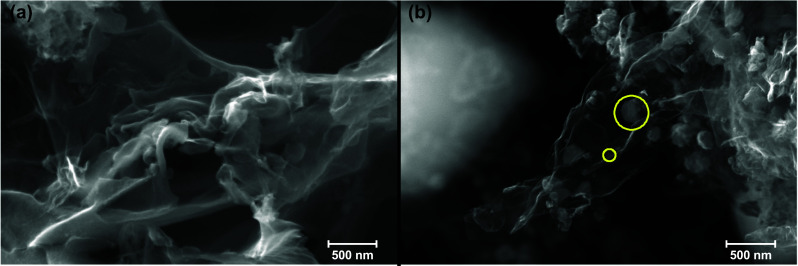
SEM images of N-GN **a** and Fe_x_O_y_/N-GN **b** The yellow circle indicates the N-GN coated iron oxide particles on N-GN

SEM images and EDS mappings were taken and given in Fig. 2 and Figure S1, respectively, for the evaluation of the morphology of the graphene samples. As depicted in Fig. 2, both N-GN and Fe_x_O_y_/N-GN samples displayed a distinct appearance characterized by a smooth, silky, and wrinkled surface, which is typical of graphene and its derivatives. Additionally, the Fe_x_O_y_/N-GN sample exhibited graphene-coated iron oxide nanoparticles and a distorted carbon surface, along with few-layered graphene structures. Moreover, the EDS mappings of both samples demonstrated a uniform distribution of carbon, nitrogen, and oxygen atoms (Figure S1). Notably, the absence of accumulations in the EDS mapping of the Fe atom for Fe_X_O_y_/N-GN (Figure S1) indicates that the iron oxide particles were evenly distributed on the graphene.

**Fig. 3:**
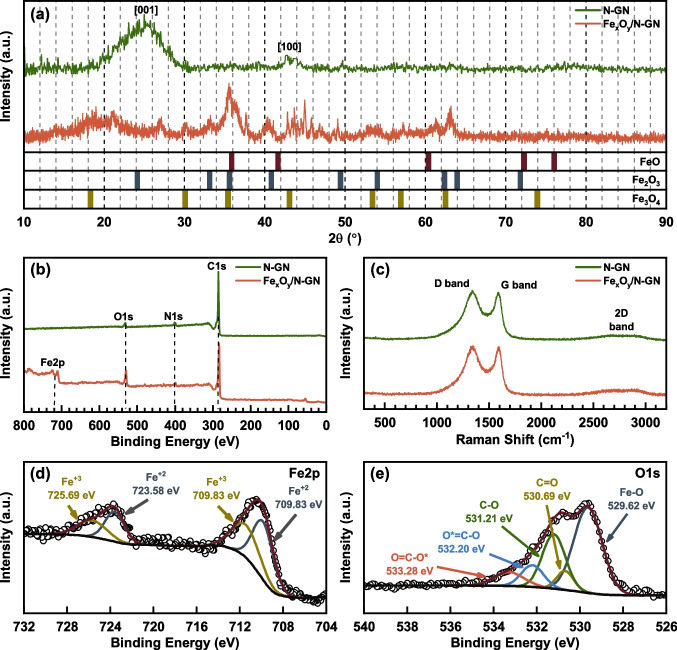
XRD **a**, XPS full elemental **b** and Raman **c** spectrum of N-GN and Fe_x_O_y_/N-GN with the detailed high-resolution spectrum of Fe2p **d** and O1s **e** of Fe_x_O_y_/N-GN

The XRD spectra of the samples were given in Fig. 3a, along with the simulated powder diffraction pattern of FeO, Fe_2_O_3_, and Fe_3_O_4_ to identify the Fe_x_O_y_ species through the crystallographic structure. The simulated patterns were calculated using the powder diffraction pattern tools of VESTA with the crystal information file (*.cif) from the Crystallography Open Database (COD); the COD ID of cif files was given in SI. The N-GN sample displayed a characteristic broad diffraction peak at 24.6°, corresponding to a layer-to-layer distance of 0.353 nm, and a peak at 43.2° attributed to unexfoliated graphene layers [21]. For Fe_x_O_y_/N-GN, the graphene peak was observed at approximately 20° with a broader shape, accompanied by a small graphite peak at 26.8°. Notably, the simulated diffraction patterns of FeO, Fe_2_O_3_, and Fe_3_O_4_ (Fig. 3a) closely matched the Fe_x_O_y_/N-GN pattern, suggesting that the iron oxide nanoparticles exist in the form of mixed Fe^2+/3+^ oxide.

Detailed information on the elemental composition of the samples and the chemical structure of the iron species can be obtained from the XPS spectra. Full survey spectra show that N-GN consists only of carbon, oxygen, and nitrogen, while Fe_x_O_y_/N-GN contains iron in addition to these, as expected (Fig. 3b). The calculated atomic percent of the elements in samples were tabulated in Table S1. The carbon ratios above 86% in both samples confirmed the presence of a less defective graphene structure. Additionally, the higher oxygen ratio of 8.76% in the Fe_x_O_y_/N-GN sample compared to N-GN (2.86%) can be attributed to the oxygen from additional iron oxide species (Fig. 3b, e). The further deconvoluted high-resolution spectra of Fe2p (Fig. 3d) revealed that the iron atoms were present in the Fe^2+^ (45%) and Fe^3+^ (55%) states, supporting the presence of mixed iron oxide species as discussed in the XRD spectrum.

The Raman spectra of N-GN and Fe_x_O_y_/N-GN were taken and given in Fig. 3c to further investigate the morphology of iron oxide nanoparticles. Herein, Raman spectra of both N-GN and Fe_x_O_y_/N-GN exhibited 3 prominent bands as D, G, and 2D bands at ~ 1340, 1585, and ~ 2800 cm^−1^, respectively. The position of the D and G bands and the shape of the 2D band indicate that both materials possess a few-layered graphene structure [22]. Additionally, the I_D_/I_G_ ratio for samples was given as Table S2 in Supporting Information along with D and G band positions. Furthermore, the absence of nanoparticle scattering peaks between 300 and 800 cm^−1^ suggests that the iron oxide nanoparticles observed in the SEM images are covered with few-layered nitrogen-doped graphene, which prevents the Raman scattering of the iron core.

Investigate the effect of the effective charge of the samples on the interaction between cellular membrane and graphene layer, Zeta potentials and particle size distributions were measured. Detailed results were given as Figure S2. Both Fe_x_O_y_/N-GN and N-GN samples showed size distribution from 200 to 1000 nm along with 21.17 mV and 3.95 mV Zeta potential respectively. These results indicate that both samples were not small enough to enter the cells directly but could interact with the negatively charged cellular membrane and wrap the individual cells.

### Cell viability and cytotoxic effect

Hepatocellular carcinoma (HCC) is a prevalent form of cancer worldwide, causing a significant number of deaths annually [23]. Unfortunately, the prognosis for HCC patients is poor, and the disease poses numerous clinical challenges for treatment. Currently, various treatment modalities, including surgery, chemotherapy, and radiation therapy, are used for HCC patients. However, these treatments often have limited success rates, highlighting the urgent need to develop more effective treatment options to improve survival rates [24]. One potential therapeutic approach for liver cancer is to enhance the cytotoxic effect of excessive iron intake due to the high iron receptor content of liver cancer cells. Several iron oxide structures, such as superparamagnetic and surface-modified nanoparticles, have been investigated in the literature. However, these iron oxide-based nanoparticles tend to agglomerate in solutions, similar to other metal-based nanoparticles, which reduces their effectiveness as drugs or results in reduced toxicity in healthy cell lines [25–27]. To address this issue, polymers and biochemicals like polyethylene glycol and chitosan have been used as surfactants or support materials [26, 28].

**Fig. 4:**
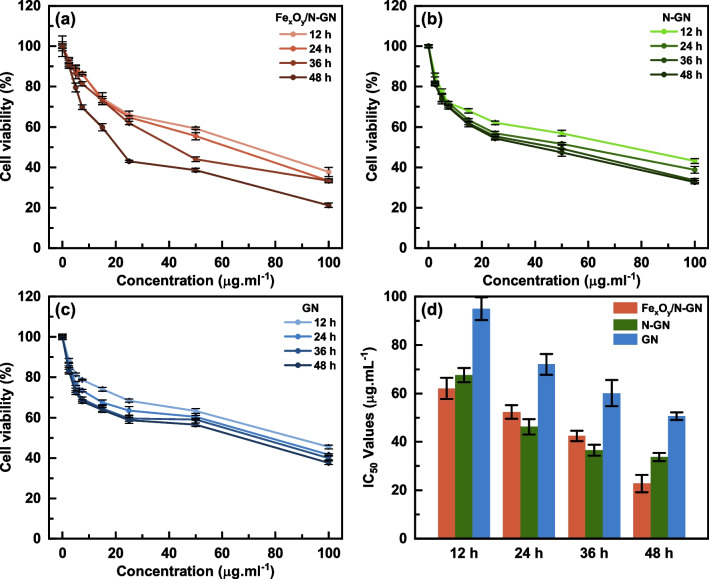
The cytotoxic effects of Fe_x_O_y_/N-GN, N-GN, and GN (2.5–100 μg/ml) for 12, 24, 36, and 48 h on BJ cells measured by CellTiter-Blue Cell Viability Assay (**a**, **b**, **c**). Results are presented as viability ratio compared with the control group (contained only the culture medium without the test-sample-untreated cells). Values are expressed as the mean of three separate experiments. Error bars represent the standard deviation (SD) of the mean from five replications (ANOVA with Tukey's test, *p* < 0.05). The IC_50_ values for each time period were given (**d**)

**Fig. 5:**
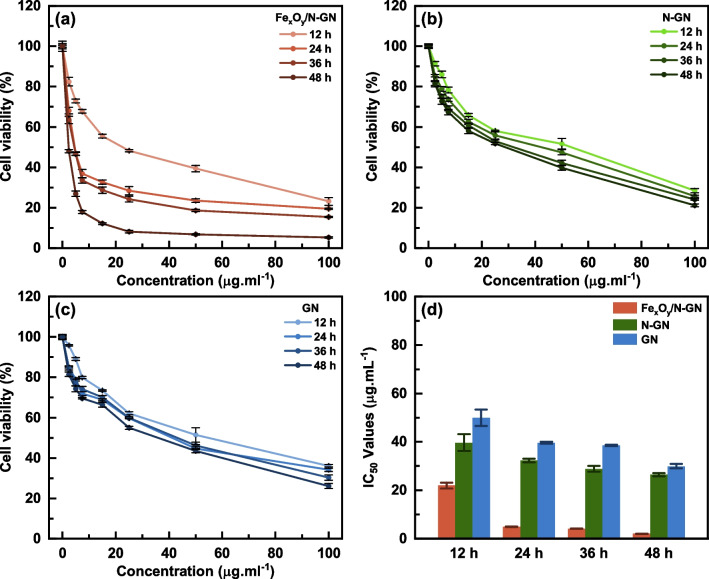
The cytotoxic effects of Fe_x_O_y_/N-GN, N-GN, and GN (2.5–100 μg/ml) for 12, 24, 36, and 48 h on HepG2 cells measured by CellTiter-Blue Cell Viability Assay (**a**, **b**, **c**). Results are presented as viability ratio compared with the control group (contained only the culture medium without the test-sample-untreated cells). Values are expressed as the mean of three separate experiments. Error bars represent the standard deviation (SD) of the mean from five replications (ANOVA with Tukey's test, *p* < 0.05). The IC_50_ values for each time period were given (**d**)

**Fig. 6:**
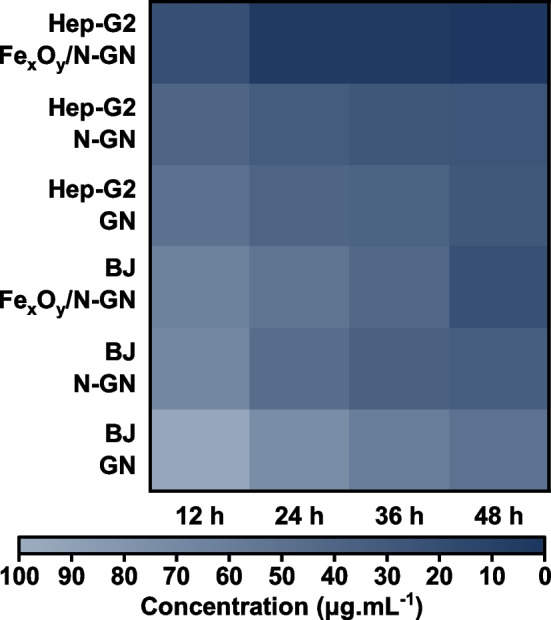
Heat map graph comparing all materials and cell lines based on the Hill model's IC_50_ (µg/mL) values

In recent years, carbon-based materials have emerged as promising candidates for various molecular biology applications, including biosensing and drug delivery, due to their chemically inert and modifiable properties [8, 29]. Among these carbon-based materials, graphene and its derivatives have garnered significant attention because of their unique 2D structure and modifiable chemical properties through heteroatom doping [30–32]. In this study, the sensitivity of liver cancer cells (HepG2) to iron atoms due to increased transferrin receptors was exploited to investigate iron-based (Fe_x_O_y_/N-GN) nanoparticles as potential drugs for hepatocellular carcinoma. Moreover, Fe_x_O_y_/N-GN was compared with other graphene materials (GN and N-GN) to observe the sole effect of iron. The effects of graphene derivatives on HepG2 and BJ cells as assessed by Cell Titer-Blue® Cell Viability Assay with different concentrations (2.5–100 μg/mL) were shown in Figs. 4 and 5. The IC_50_ values, which are the expression of dose and time-dependent inhibition of GN, N-GN, and Fe_x_O_y_/N-GN in HepG2 and BJ cells, were given in Table S3 and illustrated as a heat map in Fig. 6. Table S3 shows that IC_50_ values were calculated according to the linear and Hill functions. Based on the coefficients of determination (R^2^) for both models, it was determined that the Hill function was more appropriate for our data, and further statistical analyses were performed on these values. After 48 h of treatment, Fe_x_O_y_/N-GN was found to be 6.8 and 10.4 times more effective than N-GN and GN, respectively, in HepG2 cells. In BJ cells, Fe_x_O_y_/N-GN was 2.7 and 3.0 times more effective than N-GN and GN, respectively. Furthermore, the cytotoxic effect of Fe_x_O_y_/N-GN in HepG2 cells was six times higher than in BJ cells after 48 h of treatment. These results showed that the cytotoxic effect of all graphene derivatives in both HepG2 and BJ cells increased in a dose-dependent manner.

However, it is vital to consider the time-dependent variation of the cytotoxic effect in addition to the dose-dependent effect. In vitro reactive oxygen species (ROS) production after exposure to graphene depends on various factors related to the material and environmental conditions. These factors include size and shape, particle surface, surface charges, surface-associated chemical groups, solubility and dispersion, ions released from the cell, photo-activation, aggregation, and the mode of interaction with cells and pH of the system [33]. Therefore, to understand the effects of these variable factors in living systems, it is crucial to examine not only the dose-dependent but also the time-dependent variation of the cytotoxic effect. When the time-dependent cytotoxic effect was examined, it was observed that the cytotoxic effect of Fe_x_O_y_/N-GN on HepG2 cells significantly changed over time, while the cytotoxic effect of N-GN and GN did not. With these results, the time-dependent change in the cytotoxic effect of graphene and its derivatives in BJ cells was statistically insignificant.

Even though a vast amount of studies were conducted on the potential cytotoxic effect of graphene and its derivates, many researchers claim conflicting results and there is still ongoing debate about it. Therefore, a literature comparison is required for further analysis of the cytotoxicity of our samples. In a study conducted by Zhang et. al., it was discovered that graphene layers and single-wall carbon nanotubes (SWCNT) exhibited cytotoxic effects at low doses (~ 10 µg/ml) when exposed to the neural pheochromocytoma (PC12) cell line for 24 h [34]. In contrast to the results of the Zhang et al. study, Lasocka et al. grew the cell line directly on a graphene-coated surface to evaluate the effects of a pristine graphene monolayer on the murine fibroblast (L929) cell line [35]. CCK-8 and trypan blue assays were used to examine the cytotoxic effect of graphene on cells, and it was determined that graphene did not exhibit significant toxicity compared to the control. Additionally, phase-contrast imaging at 6, 12, and 24 h revealed no morphological changes caused by graphene. The remaining portion of the study focused on the evaluation of cytoskeleton architecture (microfilaments and microtubules), cell adhesion, and migration to the scratch-wound area using pure graphene-coated microscopic slides. Based on the obtained data, it was demonstrated that the pure graphene layer did not exhibit toxicity towards the L929 cell line.

Similarly, in a study by Rastogi et al., cells were grown on surfaces with and without graphene-coated surfaces [36]. The Hoechst 33,342 staining test results indicated that graphene did not have a cytotoxic effect on COS-7 (monkey renal epithelial cell line) cells after 24, 48, and 96 h of treatment. It was also reported that graphene did not have detectable adverse effects on mitochondrial membrane potential and morphology, autophagy levels, or induce cell stress. Consistent with these studies, the time-dependent toxicity of pure graphene on healthy cells was not statistically significant in this study, and the cell viability of the BJ cell line remained above 70% after 24 h of pure graphene application. These findings, along with the results from the studies conducted by Lasocka et al. and Rastogi et al., contribute to the evaluation of the biocompatibility of pure graphene on healthy cells.

While graphene oxide nanosheets have been widely used as drug carrier materials in numerous studies due to their high oxygen-containing functional groups and large surface area, there have been relatively few studies on the effects of nitrogen-doped graphene in cancer treatment. In a study by Baldea et al., the cytotoxic, apoptotic, and autophagic effects of three nitrogen-doped graphene samples with varying nitrogen contents (NGr-1 0.79%, NGr-2 2.56%, and NGr-3 2.33% by weight) were investigated on human endothelial (HUVEC) and colon cancer (DLD1) cells [37]. No significant cytotoxic changes were observed when examining the cytotoxic effect of nitrogen-doped graphene samples on HUVEC and DLD1 cells after 24 h. Although the study did not provide IC_50_ values for the nitrogen-doped graphene samples, it was reported that LDH and MDA levels increased more in the DLD1 cell line compared to the HUVEC cell line after 20 µg/ml graphene application. Additionally, apoptosis, as indicated by flow cytometry analysis and the expression of apoptotic proteins (NFkB, IL1a, IL6, IL10, STAT3, QH2Ax), and autophagy, as indicated by increased expression of Beclin 1, ATG16L, ATG4B, and LC3B proteins, were significantly increased in the DLD1 cell line compared to the HUVEC cell line. Considering the XRD results of all samples defined as nitrogen-doped graphene in the study conducted by Baldea et al., it was observed that the samples contained graphene oxide structures with high oxygen content. Therefore, it is not appropriate to solely attribute the cytotoxic effect observed in the HUVEC and DLD1 cell lines in this study to the presence of nitrogen atoms.

Various studies have reported the oxygen-induced toxicity of graphene oxide in both healthy and cancer cell lines. For instance, Loutfy et. al. reported changes in the morphology of HepG2 cells after 24 h of treatment with graphene oxide nanosheets (400 µg/ml) [38]. Flow cytometry analysis revealed that graphene oxide treated HepG2 cells ceased reproduction in the S phase of the cell cycle. The suppression of p53 gene expression and increased Bax gene expression also indicated that graphene oxide induced apoptotic cell death in HepG2 cells. While the dose-dependent cytotoxic effect of graphene oxide was examined in this study, the time-dependent changes in this effect were not investigated.

In this study, it was observed that the cytotoxicity of N-GN used as a delivery system for iron oxide nanoparticles, did not significantly change in HepG2 and BJ cells over time. The XPS results indicated that the nitrogen content of the nitrogen-doped graphene used as a carrier for iron oxide nanoparticles was 3.96% atomic, while the oxygen content was 2.86% atomic. Since the oxygen content of the N-GN used in this study is lower than the many values reported in the literature, the oxygen-induced toxicity in HepG2 cells was lower and could be neglected. Therefore, using low oxygen content N-GN as a carrier system for nanoparticles, such as iron oxides, in cell culture studies can be considered a safer approach in terms of cytotoxicity compared to graphene oxides.

Apart from cytotoxicity, selectivity indexes of the samples were measured. A preferred clinical agent should have a high toxic concentration but a low active concentration. In order to evaluate any anticancer activity of a sample, the cytotoxicity ratios of healthy and malignant cell lines should be determined to calculate the SI. This study obtained the SI values of graphene samples with the IC_50_ values of HepG2 and BJ cell lines at specified time periods (Table 1). In literature, a chemical compound with an SI value of 3 or higher is generally accepted as worthy of in vitro and in vivo study on cancer cell lines, and any compound with an SI value of 10 or higher has a high potential to be an anticancer drug [17, 39]. Considering the SI values obtained in this study, the N-GN and GN were found to be less than 3. In contrast, the SI value of Fe_x_O_y_/N-GN increased to more than 10.0 after 24 h.

**Table 1:** Selectivity index of samples

Sample	12 h	24 h	36 h	48 h
Fe_x_O_y_/N-GN	2.86	10.56	10.32	10.80
N-GN	1.70	1.43	1.27	1.27
GN	1.90	1.82	1.56	1.69

### Fe ion release and *8-hydroxy-2′-deoxyguanosine level analysis*

Among the graphene-based materials investigated in this study, Fe_x_O_y_/N-GN demonstrated the highest effectiveness in HepG2 cells in a dose- and time-dependent manner. Fe_x_O_y_/N-GN consists of two components: a supporting structure of N-GN and a few layered nitrogen-doped graphene-covered Fe_x_O_y_ cores. Under normal conditions, the potassium concentration is high inside the cell (140 mM), while sodium and chlorine concentrations are high outside the cell (145 mM and 110 mM, respectively), resulting in a cell membrane potential of − 70 mV. The polycationic N-GN (+ 3.93 mV) skeleton interacts with negatively charged components of the cell membrane, leading to the displacement of potassium ions on the cell surface and disruption of ionic equilibrium. This interaction increases the efflux of potassium ions from the cell and causes stress on cells as mentioned in the literature [40, 41]. However, the more polycationic Fe_x_O_y_/N-GN (+ 21.17 mV) strongly interacts with negatively charged components of the cell membrane, displacing potassium ions and causing higher cell depolarization (> − 70 mV). To restore the membrane potential and potassium equilibrium, electrogenic pumps, such as the sodium–potassium pump, expel potassium ions from the cell, leading to membrane hyperpolarization. This hyperpolarization facilitates cation uptake outside the cell to stabilize the membrane potential. As Fe_x_O_y_ is gradually released from Fe_x_O_y_/N-GN in a pH-dependent manner, Fe^2+/3+^ ions, which are abundant outside the cell, penetrate the cell through this cationic flux. As observed in this study, the increased concentration of Fe^2+/3+^ ions inside the cell disrupts the mechanism and may cause toxic effects.

**Fig. 7:**
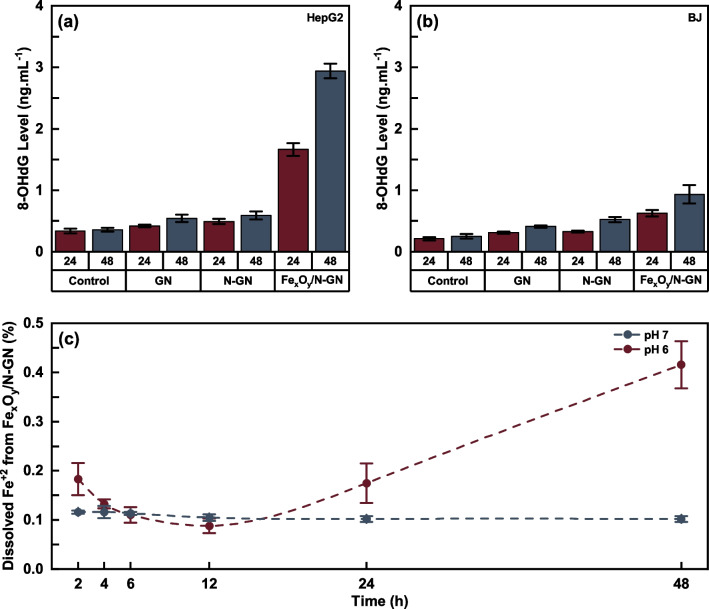
8-OHdG levels for all samples on HepG2 (**a**) and BJ (**b**) cell line with dissolved Fe ion in solution at various times from Fe_x_O_y_/N-GN (**c**)

To show whether the cytotoxic effect caused by the nanocomposites was ROS-induced, the level of 8-OHdG, a marker of ROS-induced damage in DNA, was measured. IC_50_ concentrations were selected for GN, N-GN and Fe_x_O_y_/N-GN in all measurements and the results are given in Fig. 7a and b. When the 8-OHdG levels of all samples were compared with the control group, the 8-OHdG levels of Fe_x_O_y_/N-GN nanocomposite were found to be approximately 8.17 times higher for HepG2 at 48 h. In N-GN and GN nanocomposites, this ratio was calculated as 1.6 and 1.5 times, respectively. After all nanocomposite applications, 8-OHdG levels were found to be less in Bj cells than in HepG2 cells compared to the control.

Moreover, while the relative 8-OHdG ratio of N-GN and GN did not change significantly compared to the control group, the 8-OHdG ratio of Fe_x_O_y_/N-GN was greatly increased in HepG2 cells. While 8-OHdG levels did not increase significantly in HepG2 cells after 24 and 48 h of application of GN and N-GN nanocomposites, a significant increase was observed in Fe_x_O_y_/N-GN nanocomposite at both hours. The fact that Fe_x_O_y_/N-GN showed a higher increase in 48 h application compared to 24 h supported that the effect of cytotoxic effect was based on time-dependent pH change.

At this stage, it may be beneficial to examine the pH-dependent solubilities of Fe^2+/3+^ from Fe_x_O_y_/N-GN, as determined by XRD data, in order to explain the enhanced effect observed after the 12-h mark shown in Fig. 5d. Figure S3 illustrates that the pH value of the HepG2 cell line decreased from 8.2 to 6.2 between 0 and 48 h. Previous literature reports have indicated that the solubility of Fe_x_O_y_ species within the pH range studied almost linearly as the pH decreases [42].

However, the Fe_x_O_y_/N-GN exhibited different dissolution kinetics than expected as can be seen in Fig. 7. At pH 7, a relatively small amount of Fe ion dissolved from Fe_x_O_y_/N-GN initially but after 12 h released Fe ions were reabsorbed onto graphene layers and the solution reached equilibrium. In contrast, a relatively high amount of Fe was dissolved from Fe_x_O_y_/N-GN initially and the graphene layers reabsorbed the released Fe ions again at pH 6. However, after 12 h, Fe_x_O_y_/N-GN reached the saturation level and further dissolution resulted in an increase in free Fe ion level in solution as mentioned in the literature [43]. Furthermore, after 48 h the dissolved Fe ion was only equivalent to ~ 0.45% of Fe_x_O_y_/N-GN, proving the long-term effect of Fe_x_O_y_/N-GN and the high 8-OHdG level in HepG2 cells. Consequently, the long-term anticancer effect of Fe_x_O_y_/N-GN can be attributed to the synergistic impact of the pH-dependent increase in solubility and the controlled diffusion rates of the dissolved iron species through a few layered N-GN. This pH-dependent increase in dissolution rate could result in the higher selectivity of Fe_x_O_y_/N-GN on HepG2 cells relative to the BJ cells.

For further comparison, controlled release and other metal oxide/graphene composite structures can be investigated. As an example, El-Zahed et. al. conducted a study on the cytotoxic effect of reduced graphene oxide/silver nanocomposites on Ehrlich ascites carcinoma (EAC) cells [41]. The results showed that the cytotoxic effect was dose-dependent, with approximately 87% of the EAC cells dying when incubated with 50 μg/mL of rGO/AgNC for one hour. In contrast, the cell death in normal fibroblast cells (3T3) was approximately 20%. Histological examination of kidney sections from mice treated with rGO/AgNC revealed degeneration and necrosis of the cellular epithelium in the proximal and distal renal convoluted tubules. The liver and kidney functions of the mice were also tested after treatment with rGO/AgNC, and it was found that the nano-treatment significantly increased the levels of liver enzymes ALT and AST, as well as creatinine, indicating a detrimental effect on both organs. The study by El-Zahed et al. focused on the short-term toxicity of rGO/AgNC in the HepG2 cell line, with only one-hour toxicity evaluated in healthy cells. Therefore, the effects of rGO/AgNC on healthy cells over more extended periods remain unclear.

Another study by Dorniani et al. investigated the use of graphene oxide-gallic acid as a drug delivery system in cancer treatment [44]. The release of gallic acid (GA) from the anticancer nanocomposite (GOGA) was controlled by maintaining a constant pH of 7.4 using a phosphate-buffered solution containing anions such as HPO_4_^−2^ and H_2_PO_4_^−^. Normal fibroblast cells (3T3) and liver cancer cells (HepG2) were treated with different concentrations of graphene oxide (GO), GOGA, and GA for 72 h. The IC_50_ values for HepG2 cells were 429.5, 42.9, and 38.9 μg/mL for GO nanocarrier, GOGA nanocomposite, and GA, respectively. No cytotoxic effect was observed in normal fibroblast cells, even at the highest 50 μg/mL concentration. The GOGA nanocomposite showed a cancer cell growth inhibitory effect without affecting normal cell growth. The sustained release ability of the nanocomposite may allow for reduced dosing intervals and lower exposure to large amounts of the drug.

In our study, it is important to note that the carrier system, N-GN, had low activity on cells, and no significant time-dependent cytotoxic effect was observed in both cell lines. However, the Fe_x_O_y_/N-GN system induced increased selective toxicity in cancer cells over time. This pH-dependent controlled release specifically affected HepG2 cells, which have more iron receptors than healthy fibroblast cells. Unlike the study by Dorniani et al., the active chemical release in our study was not at a fixed pH but rather examined based on dose and time, providing more detailed information. Based on the results obtained from our in vitro experiments, this study aimed to establish a cancer-specific release model that takes into account the differences between the cancer cell and healthy cell microenvironments. This model could serve as a pioneer in future in vivo tests and models.

## Conclusion

This study successfully demonstrated both pH-dependent and iron release-driven selective cytotoxic effects of nitrogen-doped graphene-coated mixed iron oxide (Fe_x_O_y_/N-GN) nanocomposites on HepG2 liver cancer cells and emphasized the utility of this nanocomposite as potential therapeutic agents. Fe_x_O_y_/N-GN has advantages over many materials in the literature in terms of its ability to utilize the pH difference of the microenvironment in cancer targeting, its controlled iron release mechanism as a result of its coating with nitrogen-doped graphene in addition to being pH sensitive, and the minimal toxicity of the support material N-GN. These properties not only enhance the efficacy of Fe_x_O_y_/N-GN as an anticancer agent but also address one of the most important challenges in cancer therapy today by minimizing secondary toxicity to non-cancerous cells.

In addition, Fe_x_O_y_/N-GN is a carbon-based inert material and more biocompatible than many tattoo inks, allowing local injection into cancer tissue. This approach not only increases the concentration of therapeutic agents at the tumor site but may also reduce systemic side effects and improve patient quality of life. The design of such clinical interventions, including controlled trials, will be critical in analyzing the efficacy and safety of nanoparticles as a cancer drug. In summary, our study lays the groundwork for Fe_x_O_y_/N-GN nanoparticles to be considered as a potential addition to cancer therapeutics. The possibility of becoming a clinically applicable drug offers hope for targeted, effective, and less toxic therapeutic strategies in the fight against cancer.

### Supplementary Information

**Additional file 1.** Supplementary materials.

## Data Availability

The authors confirm that the data supporting the findings of this study are available within the article and its supplementary materials (Additional file 1).
