# Supplementary material for: Selective cytotoxic effects of nitrogen-doped graphene coated mixed iron oxide nanoparticles on HepG2 as a new potential therapeutic approach
Source: Discov Nano. 2024 Feb 22;19(1):33. doi: 10.1186/s11671-024-03977-y (PMC10884380; doi:10.1186/s11671-024-03977-y)
Supplement: Supplementary file 1 — Additional file 1. Supplementary materials. [file 11671_2024_3977_MOESM1_ESM.doc]

**Selective Cytotoxic Effects of Nitrogen-Doped Graphene Coated Mixed Iron Oxide Nanoparticles on HepG2 as A New Potential Therapeutic Approach**

Zeynep DEMİR^a^, Berkay SUNGUR^b^, Edip BAYRAM^c^, Aysun ÖZKAN^d,*^

^a^ Department of Biology, Institute of Natural and Applied Sciences, Akdeniz University 07070, Antalya, Türkiye, 0000-0003-4014-8467

^b^ Department of Chemistry, Institute of Natural and Applied Sciences, Akdeniz University 07070, Antalya, Türkiye, 0000-0002-5567-0146

^c^ Department of Chemistry, Faculty of Science, Akdeniz University 07070, Antalya, Türkiye, 0000-0001-9800-7451

^d^ Department of Biology, Faculty of Science, Akdeniz University 07070, Antalya, Türkiye, 0000-0002-9403-3342

* Prof. Dr. Aysun Özkan, E-mail address: aozkan@akdeniz.edu.tr, Corresponding author

**Details for XPS Analysis**

The XPS analysis was carried out with Thermo Scientific K-Alpha by pipetting suspensions in 2-propanol (3 mg.ml-1) onto Si plates (1.1 × 1.1 cm) and then drying the plates in an oven at 80℃ until the entire solvent was evaporated. The samples were studied with a monochromatic anode X-ray tube with a characteristic energy of 1486.6 eV (Al Kα). A source power of 300 W was used and the pressure in the analysis chamber was below 10-9 Torr. To collect the survey, scan the following parameters were used: energy range = 1300 to 0 eV, pass energy = 200 eV, step size = 1eV. For high resolutions spectra, pass energy was changed to 50 eV.

**Details for Simulated XRD Patterns**

XRD simulations were created with Powder Diffraction Pattern tool of VESTA and Crystal Information Files from Crystallography Open Database (COD) [1–8] . COD IDs:

- FeO: 1011169
- Fe_2_O_3_: 9000139
- Fe_3_O_4_: 9006189

**Figures**


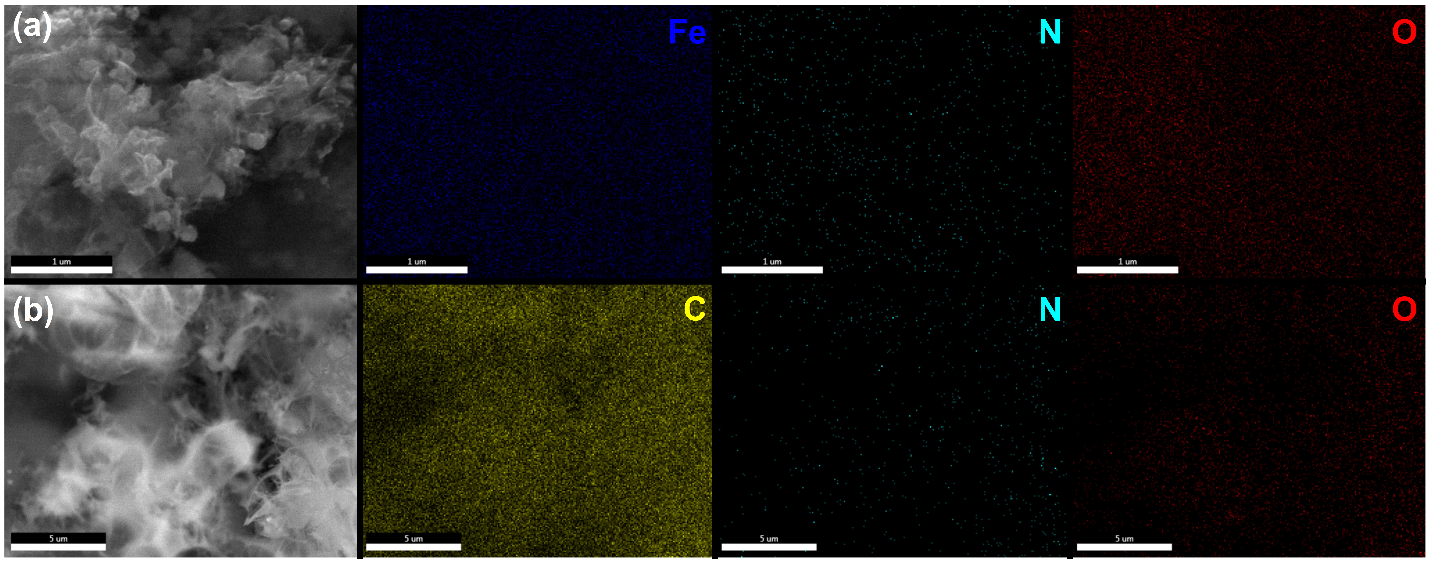


**Figure S1.** SEM elemental mappings of Fe_x_O_y_/N-GN (a) and N-GN (b)


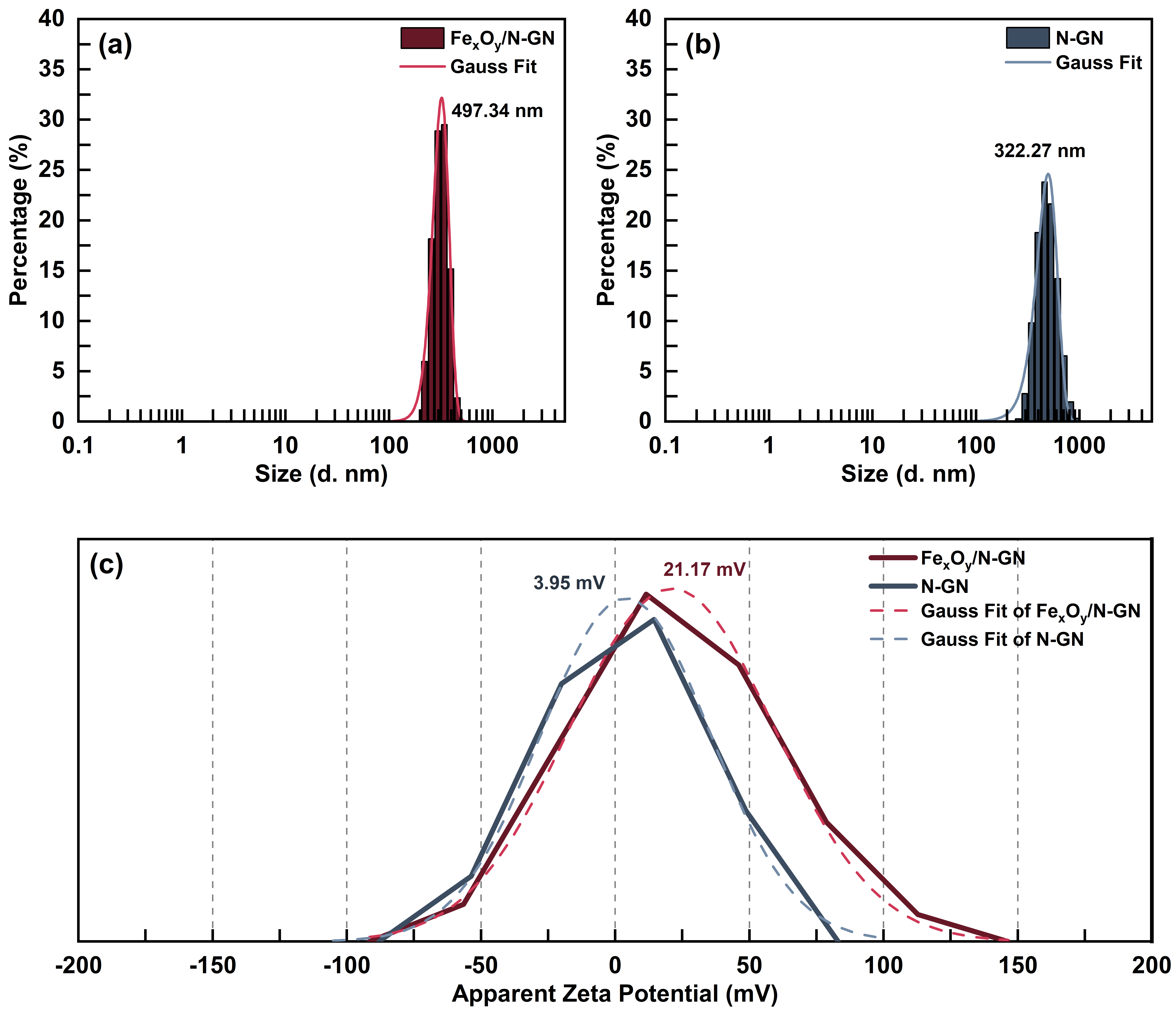


**Figure S2.** Particle size distribution of Fe_x_O_y_/N-GN (a) and N-GN (b) and Zeta potentials of both samples (c)


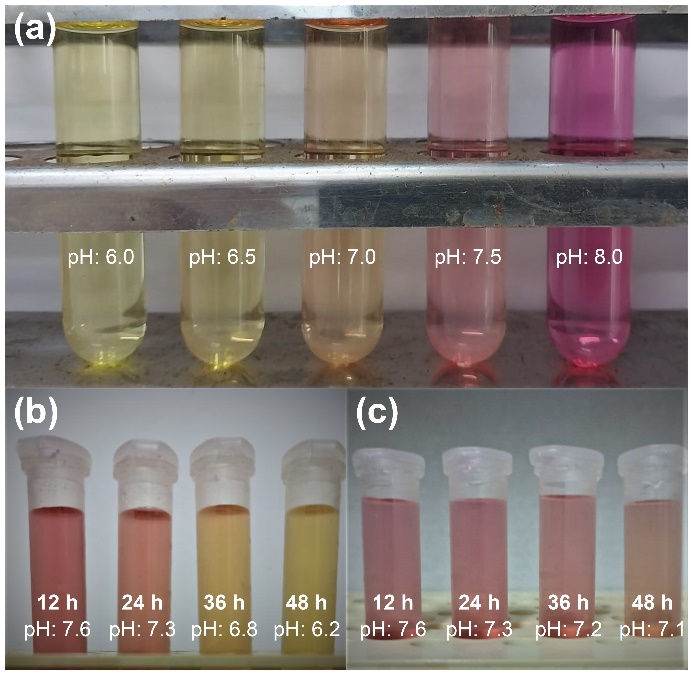


**Figure S3.** Reference Phenol Red color & pH scale in phosphate buffer solution (a) and color & pH of cell culture of HepG2 (B) and BJ (C) with respect to time

**Tables**

**Table S1.** Elemental atomic ratios of the samples

| **Sample** | **C (at. %)** | **N (at. %)** | **O (at. %)** | **Fe (at. %)** |
| --- | --- | --- | --- | --- |
| Fe_x_O_y_/N-GN | 86.4 | 3.58 | 8.76 | 1.23 |
| N-GN | 93.2 | 3.96 | 2.86 | - |

**Table S2.** Position of D and G band in Raman Spectrum and I_D_/I_G_ ratio of the samples

| **Sample** | **D band (cm^-1^)** | **G band (cm^-1^)** | **I_D_/I_G_** |
| --- | --- | --- | --- |
| Fe_x_O_y_/N-GN | 1340.43 | 1586.81 | 1.33 |
| N-GN | 1339.00 | 1580.73 | 1.34 |

**Table S3.** Calculated IC_50_ of all materials and cell lines in units of µg/mL with Hill and Linear

|  |  | ***12 h*** | | ***24 h*** | | ***36 h*** | | ***48 h*** | |
| --- | --- | --- | --- | --- | --- | --- | --- | --- | --- |
| **Cell Line** | **Material** | **IC_50_ Value**  **(Hill Model)** | **IC_50_ Value**  **(Linear Model)** | **IC_50_ Value**  **(Hill Model)** | **IC_50_ Value**  **(Linear Model)** | **IC_50_ Value**  **(Hill Model)** | **IC_50_ Value**  **(Linear Model)** | **IC_50_ Value**  **(Hill Model)** | **IC_50_ Value**  **(Linear Model)** |
| BJ | Fe_x_O_y_/N-GN | 62.08  (0.9856) | 62.72  (0.9016) | 52.36  (0.9900) | 62.63  (0.9084) | 42.50  (0.9970) | 36.92  (0.9459) | 22.78  (0.9868) | 17.79  (0.9409) |
|  | N-GN | 67.52  (0.9877) | 71.37  (0.7378) | 46.24  (0.9955) | 60.38  (0.7194) | 36.55  (0.9928) | 51.10  (0.7492) | 33.68  (0.9952) | 48.61  (0.7449) |
|  | GN | 94.99  (0.9776) | 80.92  (0.8364) | 72.03  (0.9702) | 70.54  (0.7596) | 60.14  (0.9607) | 59.01  (0.6955) | 50.60  (0.9660) | 53.58  (0.7073) |
| HepG2 | Fe_x_O_y_/N-GN | 21.95  (0.9952) | 20.17  (0.7569) | 4.96  (0.9687) | 5.28  (0.9488) | 4.12  (0.9820) | 4.74  (0.9398) | 2.11  (0.9940) | 2.95  (0.8818) |
|  | N-GN | 39.64  (0.9826) | 56.22  (0.8557) | 32.34  (0.9853) | 50.60  (0.8374) | 28.80  (0.9725) | 45.85  (0.8069) | 26.47  (0.9885) | 20.30  (0.831) |
|  | GN | 49.94  (0.9914) | 59.94  (0.8545) | 39.58  (0.9904) | 56.26  (0.8060) | 38.48  (0.9833) | 35.41  (0.8415) | 29.94  (0.9807) | 30.55  (0.7897) |

***** Model and their respected R^2^ values in parentheses.

**References**

[1] **Merkys A, Vaitkus A, Butkus J, et al.** COD::CIF::Parser : an error-correcting CIF parser for the Perl language. *J. Appl. Crystallogr.* 2016; 49; 292–301.

[2] **Quirós M, Gražulis S, Girdzijauskaitė S, et al.** Using SMILES strings for the description of chemical connectivity in the Crystallography Open Database. *J. Cheminform.* 2018; 10; 23.

[3] **Downs RT, Hall-Wallace M**. The American Mineralogist crystal structure database. *Am. Mineral.* 2003; 88; 247–50.

[4] **Gražulis S, Daškevič A, Merkys A, et al.** Crystallography Open Database (COD): an open-access collection of crystal structures and platform for world-wide collaboration. *Nucleic Acids Res.* 2012; 40; D420–7.

[5] **Vaitkus A, Merkys A, Gražulis S**. Validation of the Crystallography Open Database using the Crystallographic Information Framework. *J. Appl. Crystallogr.* 2021; 54; 661–72.

[6] **Gražulis S, Merkys A, Vaitkus A, et al.** Computing stoichiometric molecular composition from crystal structures. *J. Appl. Crystallogr.* 2015; 48; 85–91.

[7] **Gražulis S, Chateigner D, Downs RT, et al.** Crystallography Open Database – an open-access collection of crystal structures. *J. Appl. Crystallogr.* 2009; 42; 726–9.

[8] **Merkys A, Vaitkus A, Grybauskas A, et al.** Graph isomorphism-based algorithm for cross-checking chemical and crystallographic descriptions. *J. Cheminform.* 2023; 15; 25.
